# Static Expression of Major Histocompatibility Complex Genes Across Life‐Stages, Generations and Tissues in a Songbird

**DOI:** 10.1111/mec.70590

**Published:** 2026-10-16

**Authors:** Samantha Mellinger, Lars Råberg, Anna Drews, Bengt Hansson, Pavel Zehtindjiev, Dennis Hasselquist, Helena Westerdahl

**Affiliations:** ^1^ Department of Biology Lund University Lund Sweden; ^2^ Science for Life Laboratory, Department of Biology Lund University Lund Sweden; ^3^ Institute of Biodiversity and Ecosystem Research Bulgarian Academy of Sciences Sofia Bulgaria

**Keywords:** age‐related differences, consistent gene expression patterns, major histocompatibility complex (MHC), MHC Class I, passerines, relative gene expression

## Abstract

The extraordinarily polymorphic Major Histocompatibility Complex (MHC) is an iconic genomic region that has been associated with, for example, disease susceptibility, mate choice, and fitness. Both the MHC class I and II gene families have expanded considerably in songbirds (Passeriformes) compared to other bird orders, but our understanding of the functional relevance of the large number of gene copies in songbirds is hampered by lack of data on gene expression. We therefore took advantage of longitudinal data from our wild long‐term monitored population of great reed warblers *Acrocephalus arundinaceus* as well as a long‐term experiment to study patterns of MHC class I (MHC‐I) gene expression during individuals' lifetimes and across generations, and between tissues. As could be expected within a species, the expressed diversity increased linearly with genomic MHC‐I diversity. However, most MHC‐I alleles were expressed at low levels, with only a handful of alleles showing moderate to high expression in each individual. The MHC‐I expression patterns in blood were highly consistent (i) over an individual's lifetime, (ii) between parents and offspring, and (iii) among siblings, and (iv) were strongly correlated with expression in other tissues. The moderately to highly expressed MHC‐I genes likely play a key role in antigen presentation in adaptive immunity and thus comprise primary targets for studies of host‐pathogen coevolution.

## 1 Introduction

The enormous diversity of T‐cells is the foundation of the adaptive immune system in jawed vertebrates (Murphy and Weaver 2017). The T‐cell mediated adaptive immune response against pathogens relies on successful antigen presentation by Major Histocompatibility Complex (MHC) molecules. MHC molecules present peptides (antigens) on the cell surface, whereafter T‐cells interact with the MHC‐antigen‐complex and can become activated (Neefjes et al. 2011). MHC Class I molecules (MHC‐I) are expressed on all nucleated cells and present cytosolic peptides (i.e., self‐peptides and peptides from intracellular pathogens) to cytotoxic T cells (Murphy and Weaver 2017). MHC‐I genes have undergone a substantial expansion in songbirds (Aves: Passeriformes) compared with other birds (Minias et al. 2018; He et al. 2021; O'Connor et al. 2016; Biedrzycka et al. 2017) and mammals (Kumánovics et al. 2003; Day et al. 2024). For example, chicken and human possess only two and three (classical) MHC‐I genes, respectively, whereas many songbirds carry more than 20 genes (Kaufman et al. 1999; Shiina et al. 2009; O'Connor et al. 2019). Despite this remarkable expansion, the functional relevance of the extensive MHC‐I gene repertoire in songbirds remains largely unknown.

The MHC genes are the most polymorphic genes known in vertebrates (as indicated by the number of different MHC alleles per gene in a population), and associations between MHC‐I alleles and resistance to infectious diseases have been reported in a wide range of different species (Savage and Zamudio 2011; Martin and Carrington 2013; Wallny et al. 2006; Omar and Schat 1996). Pathogen‐mediated balancing selection is thought to be the main driving force for the maintenance of the high MHC polymorphism (Radwan et al. 2020). In songbirds, MHC‐I has been associated with qualitative and quantitative resistance to avian malaria infections in a handful of species (Bonneaud et al. 2006; Westerdahl 2007; Loiseau et al. 2011; Sepil et al. 2013; Westerdahl et al. 2012). On a macroevolutionary scale, songbird species in pathogen‐rich areas have higher MHC diversity (number of different MHC alleles per individual) than species in pathogen‐sparse areas, indicating a selective advantage of high MHC diversity (O'Connor et al. 2020, 2018).

The proportion of expressed MHC‐I alleles varies greatly between taxonomic families in songbirds, whereas the expression is rather similar among species within families. Some songbird species express all MHC‐I alleles in the genome, whereas other species express less than 50% (O'Connor and Westerdahl 2021). The relative expression of the different MHC‐I alleles within an individual, from now on called ‘relative expression’, also varies considerably between taxonomic families. Some songbird species express one to two MHC‐I alleles moderately to highly and many alleles lowly, whereas other species express as much as 50% of their MHC‐I alleles moderately to highly (Drews et al. 2017; Drews and Westerdahl 2019; Watson et al. 2024). A critical, but as yet little investigated question is how stable the relative MHC‐I expression is over time, both within and across generations. A highly stable MHC‐I expression pattern, where alleles at specific genes are either always highly or lowly expressed, would suggest that the different MHC‐I gene copies have different roles in the immune defense. For example, the chicken ( *Gallus gallus* , Galliformes) often has one highly and one lowly expressed MHC‐I gene copy, and only the highly expressed gene is assumed to encode MHC‐I molecules of central importance for adaptive immunity (Wallny et al. 2006).

In this study, we examine MHC‐I gene expression patterns (the relative expression of the different MHC‐I alleles within an individual) using data from a long‐term monitored population of great reed warblers *Acrocephalus arundinaceus* in south Central Sweden (> 40 years; [Hasselquist 1998; Tarka et al. 2015; Roved et al. 2018]), and taking advantage of samples collected in an experiment on caged great reed warblers. Our analyses focus on three aspects of MHC‐I expression: (1) variation within individuals during their lifetime (repeated sampling, between life‐stages and breeding seasons), (2) variation across generations (between parents and offspring, that is, the inheritance pattern of relative expression), and (3) variation across different tissue types. The MHC‐I genes in the great reed warbler are organized as tightly linked paralogs (Westerdahl et al. 2022), inherited as individual MHC‐I haplotypes, although with considerable gene copy number variation between haplotypes (Westerdahl et al. 2004; Roved et al. 2022). In our study population, pedigrees are available and blood samples for DNA (gDNA) and RNA (cDNA) analyses have been collected from parents and their offspring. We genotyped MHC‐I in gDNA and cDNA and measured the gene expression pattern in parents and offspring using high‐throughput amplicon sequencing (HTS). We used pedigree information to infer parental haplotypes and compared the MHC‐I gene expression pattern per haplotype in parents and offsping.

## 2 Results

### 2.1 Highly Consistent MHC‐I Gene Expression Patterns Both Between Life Stages and During Adulthood

We genotyped and analysed the relative MHC‐I gene expression in blood in 17 great reed warbler families, including 31 parents (15 males and 16 females) and 48 nestlings (1–4 per brood, on average 2.8) (Figure 1a, Table S1). Across all samples, we detected 184 different MHC‐I alleles (exon 3) in gDNA and 159 of these also in cDNA. The expressed MHC‐I diversity (cDNA) increased linearly with MHC‐I diversity in gDNA (*N* = 31, *r* ^2^ = 0.70, F_(1,29)_ = 70.56, *p* < 0.001, quadratic term: *p* = 0.71; Figure 1b). On average, individuals expressed 11.3 (SD = 5.2; range 2–23) cDNA alleles, and typically less than a handful of these were moderately to highly expressed, whereas most alleles were lowly expressed (Figure 1c).

**FIGURE 1:**
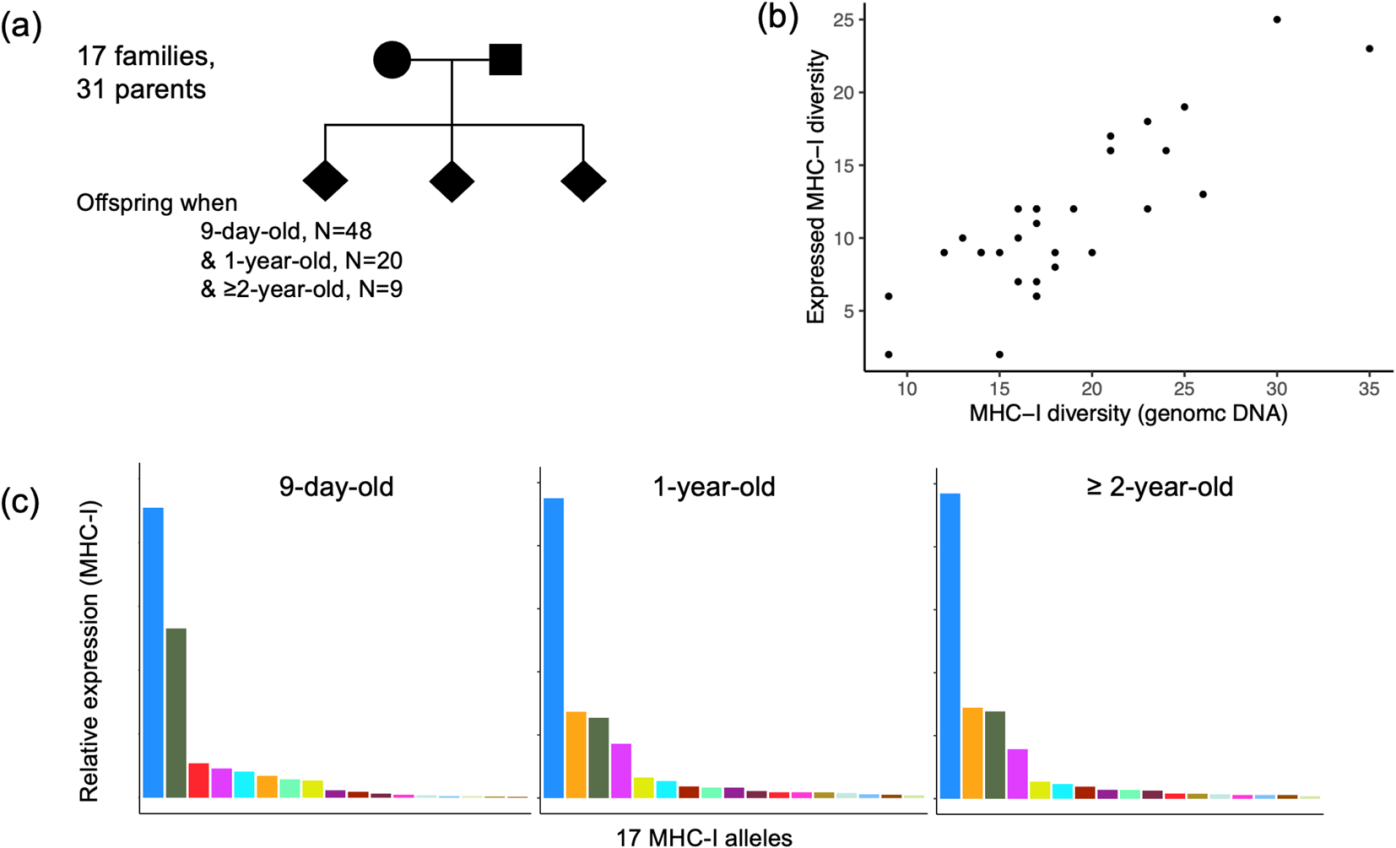
(a) MHC‐I expression was investigated in parents (15 males and 16 females) and offspring (1–4; mean 2.8 nestlings per brood) in 17 great reed warbler families. One nestling per brood was sampled at three time points: As 9‐day‐old, 1‐year‐old and ≥ 2‐year old. (b) The number of expressed alleles (cDNA) in relation to the number of alleles in the genome in the 31 parents (four points are overlapping). (c) Example of the relative gene expression of the MHC‐I alleles of one offspring when 9‐days, 1‐year and ≥ 2‐year old, respectively.

To investigate if the relative expression of different MHC‐I alleles was consistent during the lifetime of an individual, we calculated Spearman rank correlations between time points (nestling, 1‐year‐old and ≥ 2‐year‐old birds; *N* = 9 individuals). The relative MHC‐I gene expression pattern in an individual was highly consistent over its life, both between life‐stages (nestling vs. 1‐year‐old) and between adult ages (1‐ vs. ≥ 2‐year‐old). The average correlation (r_S_) of relative expression was 0.85 (*N* = 9, range 0.76–0.93) between life‐stages and 0.94 (*N* = 9, range 0.82–1.0; Figure 2a,b) between ages at adulthood. The correlation between different ages at adulthood (1‐year‐old vs. ≥ 2‐year‐old) was significantly higher than between life stages (9‐day‐old nestlings vs. 1‐year‐old; paired sample Wilcoxon test, *N* = 9, V = 5, *p* = 0.039; Figure 2*c*).

**FIGURE 2:**
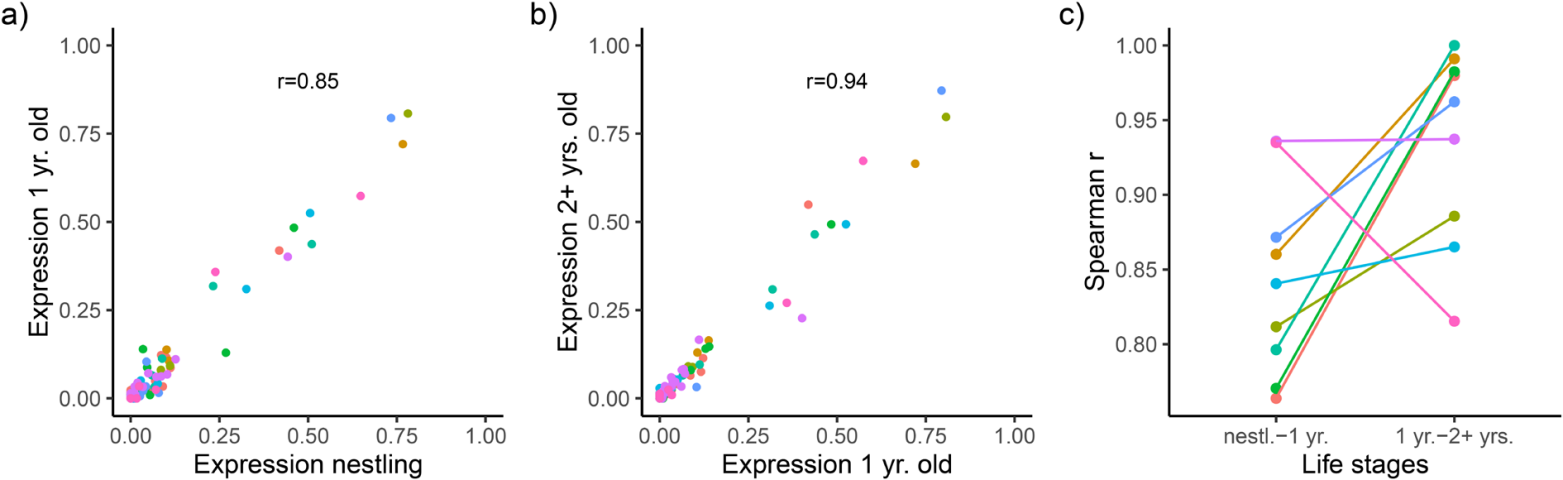
Correlation of expression of MHC‐I alleles between life stages in nine individuals (9‐day‐old nestling vs. 1‐year‐old) and between years in adults (1‐year‐old vs. ≥ 2‐year‐old). (a) 9‐day‐old nestling vs. 1‐year‐old, (b) 1‐year‐old vs. ≥ 2‐year‐old, and (c) Pairwise comparison of correlation coefficient (r) between life stages (9‐day‐old nestling vs. 1‐year‐old) and during adulthood (1‐year‐old vs. ≥ 2‐year‐old) in nine individuals.

### 2.2 Inheritance of MHC‐I Gene Expression Patterns

In the paragraph above we investigated the relative expression of all MHC‐I alleles in individuals (based on the MHC genotype). In Sections 2.2 and 2.3 we instead focus on the relative expression of each MHC‐I allele on specific haplotypes, as inferred from inheritance of alleles in 17 families. Twentyeight maternal and 28 paternal haplotypes were identified based on the segregation of alleles (Figure 1a, Table S1).

The relative expression of the MHC‐I alleles in one maternal and one paternal MHC‐I haplotype in one nestling per clutch was plotted against the relative expression of the alleles in the same haplotype in the respective parent (Figure 3). Relative expression (log_10_‐transformed) of MHC‐I alleles on maternal haplotypes was highly similar between the mother and her nestlings (fixed effect slope = 0.896 ± 0.060, *N* = 11, *p* < 0.001, Figure 3a). Similar results were found when using data from when offspring were 1‐year‐old (fixed effect slope = 0.967 ± 0.045, *N* = 11, *p* < 0.001, Figure 3b). Likewise, the relative expression of MHC‐I alleles on paternal haplotypes was highly similar between fathers and their nestlings (fixed effect slope = 0.873 ± 0.065, *N* = 11, *p* < 0.001, Figure 3c). Similar results were found when using data from when offspring were 1‐year‐old (fixed effect slope = 0.974 ± 0.041, *N* = 11, *p* < 0.001, Figure 3d). There was no significant variation in intercept or slope among families, except for expression of paternal haplotypes in 1‐year‐old offspring (slope: *p* = 0.024). The inheritance of the MHC‐I gene expression pattern is high as shown by strong associations between relative expression per haplotype in offspring and parents.

**FIGURE 3:**
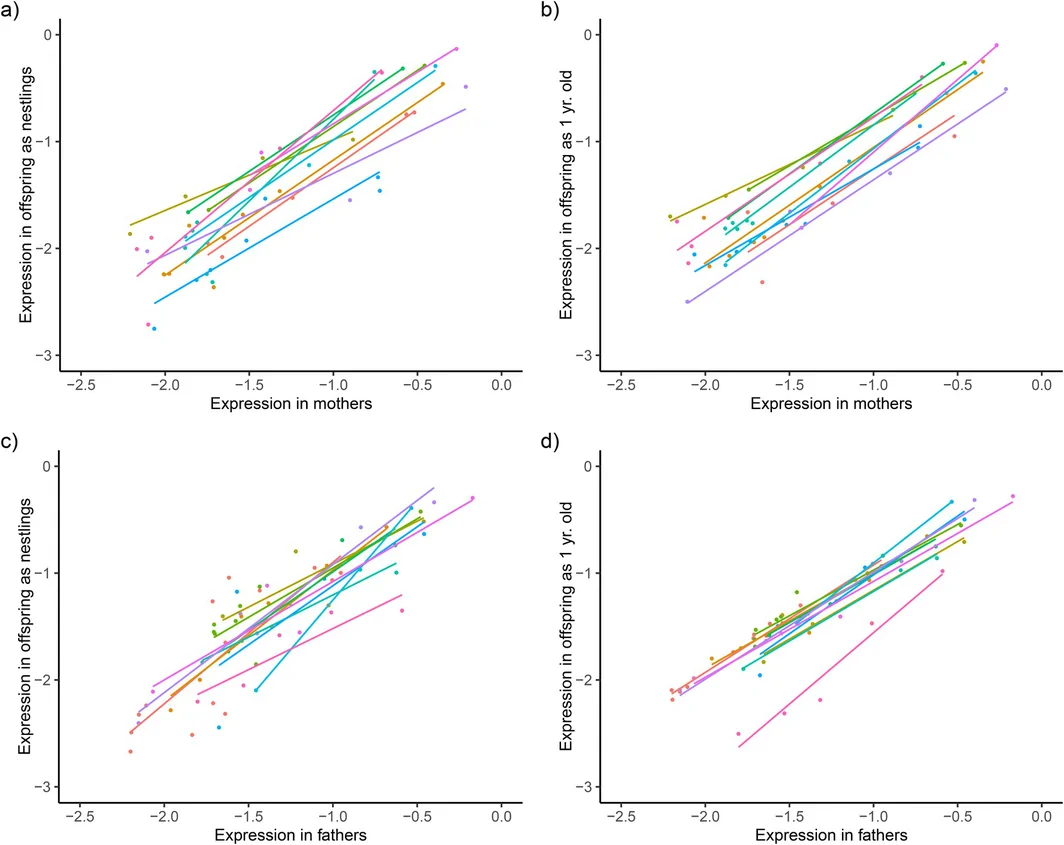
Inheritance of MHC‐I gene expression in (a) Maternal and nestling haplotypes, (b) Maternal and 1‐year‐old bird haplotypes (one nestling per clutch, *N* = 11 haplotypes), (c) Paternal and nestling haplotypes and (d) Paternal and 1‐year‐old bird haplotypes (one nestling per clutch, *N* = 11 haplotypes).

### 2.3 MHC‐I Gene Expression Patterns in Offspring With Identical and Different Parental MHC‐I Haplotype Background

To test for effects of genetic background on the relative expression of MHC‐I alleles, we compared relative expression in siblings sharing one parental haplotype (maternal or paternal) in combination with either identical or different MHC‐I haplotype background. If the haplotype background influences relative expression in the shared parental haplotype, we expect the slope (fixed effect) to be lower when comparing siblings having different haplotype backgrounds compared with siblings sharing the same parental haplotype background. Relative expression (log^10^‐transformed) of MHC‐I alleles on maternal haplotypes was highly similar in siblings sharing the same paternal haplotype background (fixed effect slope = 0.979 ± 0.084, *N* = 6, *p* < 0.001, Figure 4a). Similar results were found for relative MHC‐I expression in maternal haplotypes in siblings with different paternal haplotype backgrounds (fixed effect slope = 0.903 ± 0.097, *N* = 7, *p* < 0.001, Figure 4b). Relative expression in paternal haplotypes was highly similar in siblings sharing the same maternal haplotype background (fixed effect slope = 0.967 ± 0.069, *N* = 7, *p* < 0.001, Figure 4c). Again, the same pattern was seen for siblings with different maternal haplotype backgrounds (fixed effect slope = 0.884 ± 0.089, *N* = 4, *p* < 0.001, Figure 4d). There was no significant variation in intercept or slope among families (*p* > 0.14). Since the standard errors of the fixed effects slopes overlapped between analyses of offspring that shared or did not share background haplotypes, there is no significant support for MHC haplotype background affecting relative expression of MHC‐I alleles.

**FIGURE 4:**
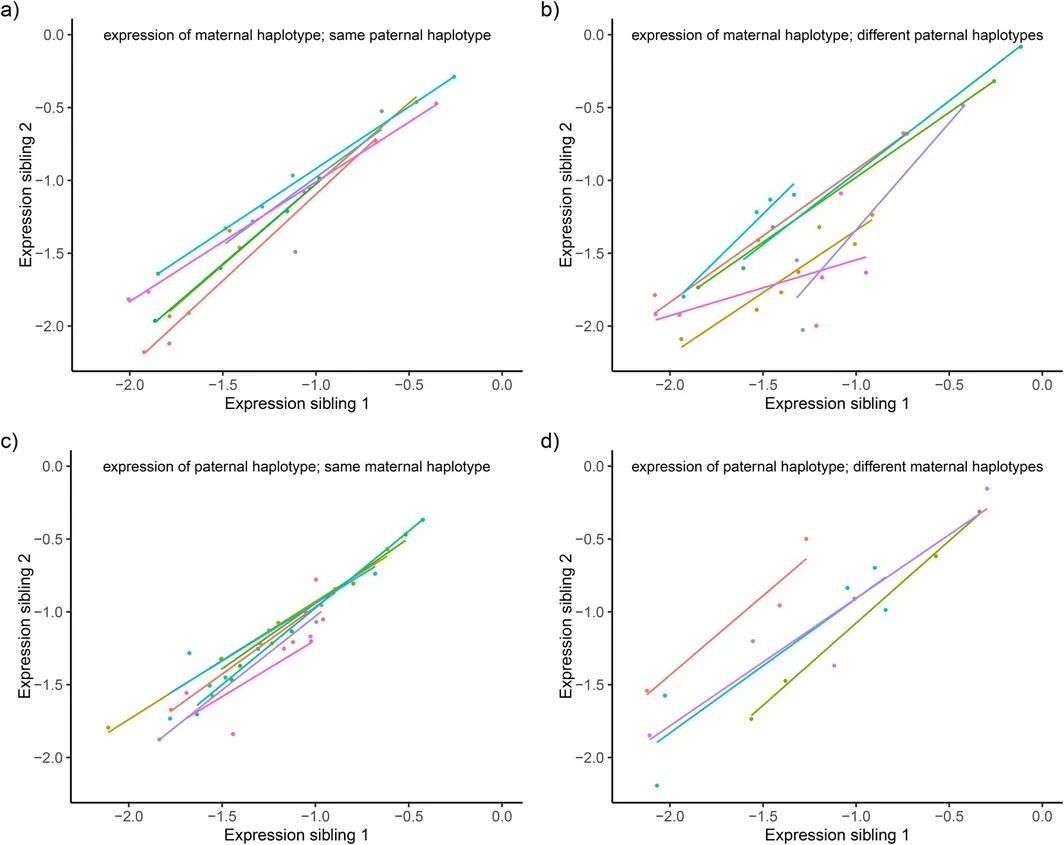
Similarity in MHC‐I gene expression for (a) the maternal haplotype in siblings with identical parental MHC‐I haplotypes (*N* = 6 haplotypes, 6 families), (b) the maternal haplotype in siblings that have identical maternal but different paternal haplotypes (*N* = 7 haplotypes, 6 families), (c) the paternal haplotype in siblings that have identical parental haplotypes (*N* = 7 haplotypes, 7 families), and (d) the paternal haplotype in siblings that have identical paternal but different maternal haplotypes (*N* = 4 haplotypes, 3 families).

### 2.4 Correlation in MHC‐I Gene Expression Patterns Between Tissues Within Individuals

The relative expression of each MHC‐I allele in an individual was highly correlated between the four investigated tissues (blood, liver, lung and intestine), although mean correlations were stronger among liver, lung and intestine (r_S_ = 0.91–0.94) than in comparisons with blood (r_S_ = 0.78–0.81; Figure 5). However, note that highly expressed alleles remained highly expressed across all four tissues (Figure 5).

**FIGURE 5:**
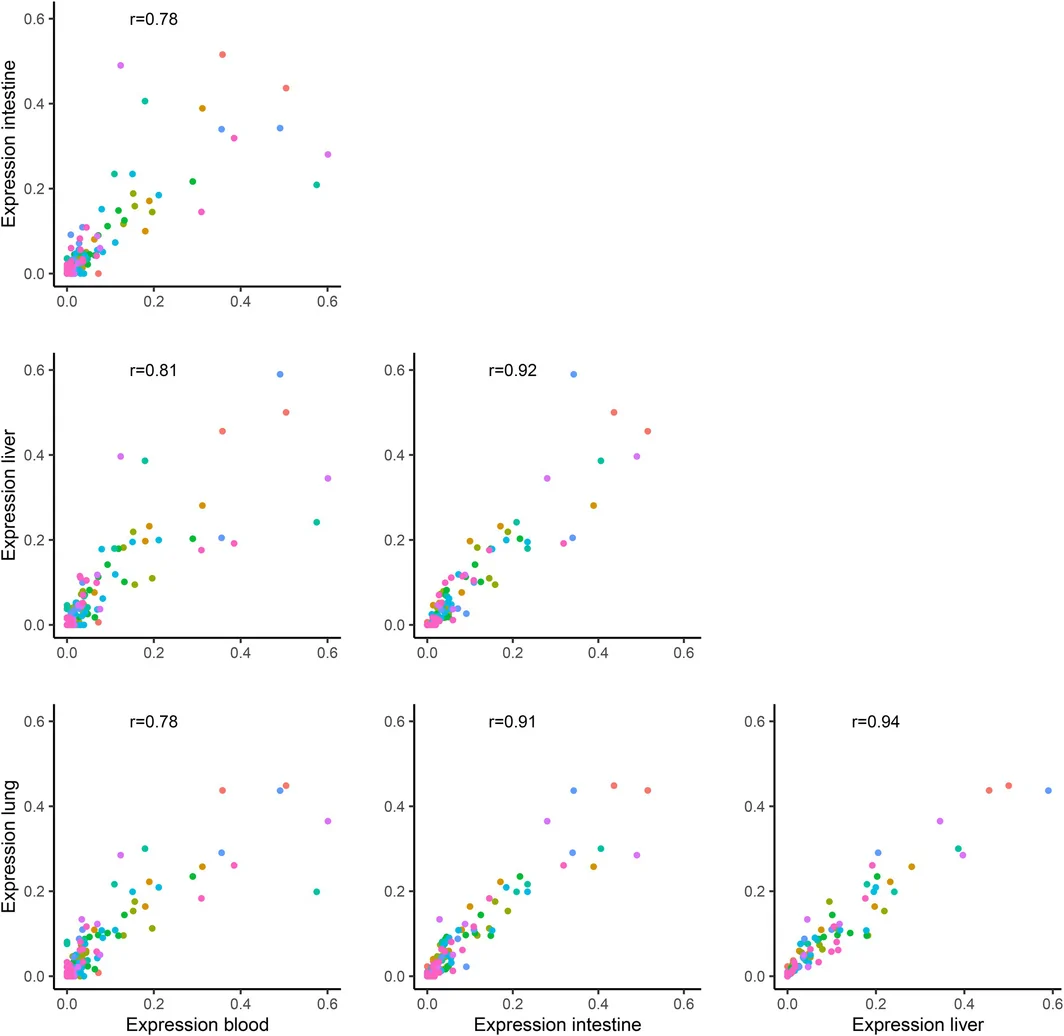
Correlation in MHC‐I gene expression between four different tissues in nine control individuals from an experiment. Each data point represents one MHC‐I allele in one individual, colour coded by individual. Correlations are Spearman rank correlations (the mean of the correlations for each individual).

## 3 Discussion

We found that the pattern of MHC‐I gene expression in blood (measured as the relative read depth of each transcribed MHC‐I allele within an individual) in great reed warblers is remarkably consistent both during the lifetime of an individual as well as between generations, that is, from parents to offspring. Moreover, the MHC‐I gene expression profile in blood was highly correlated with that in the other investigated tissues (lung, liver and intestine), indicating that blood provides a reliable representation of tissue‐wide MHC‐I expression. Although the correlations were slightly stronger between the three tissues lung, liver and intestine compared with blood, the overall consistency supports the use of blood‐derived data in expression analyses. MHC‐I molecules are ubiquitously expressed on nucleated cells and continuously present self‐peptides on the cell surface (Neefjes et al. 2011; Jongsma et al. 2019). The static nature of the relative expression levels among individual MHC‐I alleles over individuals' lifetime, across generations and between tissues shows that rather few MHC‐I genes are moderately to highly expressed while most genes are lowly expressed. Chicken, the avian model species for MHC work, has two classical MHC‐I gene copies that are highly and lowly expressed, respectively, and these gene copies encode MHC‐I molecules with different functions/roles in the immune defence (Wallny et al. 2006; Koch et al. 2007; Chappell et al. 2015). We envision a similar division of roles in the immune defence in the great reed warbler and hypothesize that the most highly expressed MHC‐I gene(s) is particularly important for antigen presentation and therefore the likely target of pathogen‐mediated balancing selection.

There were some subtle differences in the MHC‐I gene expression profiles. For example, the expression profiles within individuals were more similar from 1 year to the next within adulthood (1‐year‐old vs. ≥ 2‐year‐old birds) than between life stages (9‐day‐old nestling and 1‐year‐old adult, Figure 2). Moreover, the inheritance of MHC‐I gene expression patterns tended to be higher in the comparisons between 1‐year‐old offspring and their parents than in the comparisons between life stages (9‐day‐old nestlings and their parents, Figure 3), although not significantly so (as judged from overlapping standard errors). A possible explanation for these patterns could be that the composition of blood cells varies with life stage, and that different blood cell types express their MHC‐I gene copies slightly differently. In line with this reasoning, the composition of leukocytes is known to change during the nestling and fledgling periods and to differ between the fledgling and the adult life stage in other songbird species (Muriel et al. 2021). Moreover, the adaptive immune system, that relies on antigen recognition by lymphocytes, is for instance only fully developed in adults (Murphy and Weaver 2017; Killpack et al. 2013).

Siblings with two identical MHC‐I haplotypes could be expected to show more similar MHC‐I gene expression patterns than siblings only sharing one MHC‐I haplotype. However, we did not find support for such a difference as there were no statistically significant differences in slopes between offspring that had the same or different background haplotypes. Finally, we did not observe any differences in the MHC‐I gene expression pattern related to maternal and paternal inheritance or related to maternal and paternal haplotypes in the MHC‐I gene expression comparison between siblings. It should be noted that some of the analyses on relative MHC‐I expression on haplotypes were based on relatively small sample sizes and therefore had limited statistical power. Though, as mentioned above, the MHC‐I gene expression pattern in great reed warblers is remarkably consistent in all our comparisons.

Great reed warbler individuals in our study population in Lake Kvismaren express on average 11.3 ± 5.2 (SE) MHC‐I alleles per individual and less than a handful of these alleles are moderately to highly expressed, while the remaining alleles are lowly expressed. The most highly expressed MHC‐I allele in each individual was on average represented by more than 50% of the total read count in cDNA, although there was considerable variation in the total read count of the most highly expressed MHC‐I allele among individuals (16%–97%). Six of the thirty‐one parental individuals shared their most highly expressed MHC‐I allele with one or two other parental individuals, and the expression of these MHC‐I alleles was similarly high in all individuals. Moreover, the most highly expressed allele in the MHC‐I gene expression profiles remained the most highly expressed allele in the nine individuals that were sampled three times during their lifetime (as 9‐day‐old nestling, and as 1‐year‐old and 2‐year‐old adults); hence, it is clear that a specific MHC‐I allele is always the one that remains most highly expressed during the lifetime of an individual. From previous work, we know MHC‐I genes are tandemly organized in the genome, with short intergenic distances, and that gene duplication events likely have been frequent during their expansion in great reed warblers (Westerdahl et al. 2022). As the relative expression differs substantially between the different MHC‐I alleles it is likely that expression is regulated separately for each MHC‐I gene.

If the few moderately to highly expressed MHC‐I alleles are the ones that are of primary importance in the adaptive immune response (as in chickens), why then do songbirds maintain such large numbers of transcribed MHC‐I alleles in open reading‐frame? Several comparative studies have reported associations between MHC diversity and pathogen diversity (O'Connor et al. 2020, 2018), suggesting that high MHC diversity is favoured by selection in pathogen‐rich areas. A selective advantage of high MHC‐I diversity in pathogen‐rich areas implies that not only the moderately to highly expressed alleles are important in the adaptive immune response, but also that the lowly expressed MHC‐I alleles have a function in the immune response. The great reed warbler is a long‐distance migrant that spends the non‐breeding season in sub‐Saharan Africa where it is exposed to a wide range of pathogens (Bensch et al. 2007; Koleček et al. 2016; Malmiga et al. 2026; Bordes et al. 2011; Guernier et al. 2004; Nunn et al. 2005). Indeed, many of the malaria parasites carried by the great reed warblers in Lake Kvismaren are transmitted in sub‐Saharan Africa (Asghar, Hasselquist, et al. 2015; Bensch et al. 2007). In contrast, pathogen‐mediated selection is expected to be lower during the breeding season in the Northern Palearctic. However, the present selection from pathogens may not solely explain the MHC diversity we observe today, because the phylogenetic history and relatedness among species contribute to the diversity (O'Connor et al. 2016, 2020, 2018). All Acrocephalus warblers seem to have many MHC‐I alleles and most of the species where MHC‐I has been genotyped are long‐distance migrants ( *A. scirpaceus* and *A. schoenobaenus* ) (Biedrzycka et al. 2017; O'Connor and Westerdahl 2021; Halupka et al. 2026). Interestingly, the single African resident Acrocephalus species genotyped so far ( *A. gracilirostris* ) has the highest MHC‐I diversity, whereas the single resident European Acrocephalus species ( *A. melanopogon* ) has the lowest (O'Connor et al. 2020, 2018).

The numerous lowly expressed MHC‐I alleles in the great reed warbler most likely have a somewhat different role in the immune system than the highly expressed genes, with at least some of them representing so called non‐classical MHC‐I genes. Non‐classical MHC‐I genes have lower and more tissue‐specific expression than classical MHC‐I genes, and are also less polymorphic (Shiina et al. 2009). In the chicken, the non‐classical MHC‐I genes are physically separated from the classical MHC‐I genes on chromosome 16 (Afanassieff et al. 2001). In songbirds, including the great reed warbler, it is not yet clear which MHC‐I alleles are classical and which are non‐classical, because it is challenging to assemble the extended and repeat‐rich MHC region in songbirds and to properly investigate polymorphism per MHC‐I gene copy (Westerdahl et al. 2022; He et al. 2022). Some of the lowly expressed genes in the great reed warbler are found next to highly expressed MHC‐I genes (Westerdahl et al. 2022, 1999), contrary to the organization of classical and non‐classical genes in the chicken genome. Regardless of the function of lowly expressed MHC‐I alleles in the great reed warbler (i.e., whether they are classical or non‐classical), a potential additional benefit would be that they can act as a reservoir (or library) of MHC‐I allele variants which can contribute with new genetic variation to the highly expressed alleles via gene conversion, a mechanism frequently reported to occur in the evolution of MHC genes (Spurgin et al. 2011; Edwards and Hedrick 1998; Minias et al. 2016).

We conclude that the MHC‐I gene expression pattern in great reed warblers is highly static: the most highly expressed MHC alleles remain the most highly expressed (i) during the lifetime of an individual, (ii) from parent to offspring, (iii) in siblings with two identical MHC‐I haplotypes and (iv) in siblings with one identical and one different MHC‐I haplotype.

## 4 Material and Methods

### 4.1 Study Species and Sample Collection From the Study Population at Lake Kvismaren

The great reed warbler is a long‐distance migratory songbird that breeds in Europe and western Asia and winters in sub‐Saharan Africa (Koleček et al. 2016). It has a socially polygynous mating system where males can form pair bonds with up to at least four females simultaneously (Asghar, Bensch, et al. 2015; Westerdahl et al. 2000). Sampled birds are part of a long‐term monitored (since 1984) wild population breeding at Lake Kvismaren in southern Central Sweden and for which complete family records are available (Hansson et al. 2007; Tarka et al. 2015; Asghar, Bensch, et al. 2015). We selected 17 nests (31 parental individuals, 15 males and 16 females) where at least one nestling came back as adult to the breeding area in 2015 to 2019. Out of 48 hatched nestlings, 20 nestlings came back once after wintering in Africa (i.e., when 1‐year old) and nine of these nestlings came back at least twice (i.e., when ≥ 2‐year‐old). Blood samples were collected during the breeding seasons, that is, early May to late July, and were taken from the brachial vein on adults caught in mist‐net and from the tarsus vein on offspring when still in the nest at an age of 8–10 days. Approximatively 20–80 μL of blood was suspended in SET buffer (150 mM NaCl, 50 mM TRIS, 1 mM EDTA, pH 8.0) and kept at −20°C for 1–3 months and then stored at −40°C for DNA extraction, and 20–30 μL of blood was suspended in RNA later (RNAprotect Tissue Reagent, QIAGEN) and kept at −20°C for 1–3 months and then stored at −40°C until RNA extraction.

### 4.2 Collection of Tissue Samples

During 2018–2020 two avian malaria infection experiments were carried out on great reed warblers caught in the wild close to the Kalimok Research Station in Bulgaria (where this species is numerous). In the ‘long‐term’ experiment (2018–2020), 19 juvenile great reed warblers were caught in the wild in July–August 2018. These birds were taken indoors and held in cages in groups of up to 10 individuals with ad libitum access to food and water. In August–September 2018, these great reed warblers were either inoculated with uninfected great reed warbler blood (controls) or with great reed warbler blood containing malaria parasites (malaria infected individuals). In the ‘short‐term’ experiment (2019–2020), another 19 juvenile great reed warblers were caught in the wild in July–September 2019 and then held in indoor cages in groups of up to 10 individuals with ad libitum access to food and water. In September 2019, these great reed warblers were inoculated with either malaria‐infected or uninfected blood from donor great reed warblers. The great reed warblers from both the long‐term and the short‐term experiments were then kept in captivity and blood sampled regularly (to be used for DNA and RNA extraction) until September 2020 (‘long‐term’ experiment) or January 2020 (‘short‐term’ experiment). At the end of the experiments, birds were euthanized by decapitation and samples from numerous tissues collected in RNA later (RNAprotect Tissue Reagent, QIAGEN) and stored at –20°C and then later at Lund University at –40°C until RNA extraction. For the present study, we used the control individuals (that had not been infected with avian malaria), selecting five individuals from the ‘long‐term’ and four individuals from the ‘short‐term’ experiment. From these individuals, we analysed RNA samples from four different tissue types: blood, lung, liver and small intestine.

### 4.3 gDNA and RNA Extraction

Total genomic DNA (gDNA) was extracted from blood preserved in SET buffer using standard phenol‐chloroform extraction protocol and resuspended in 1X TE buffer (10 mM Tris, 1 mM EDTA, pH 8.0) for long‐term storage at −40°C.

Total RNA from blood was extracted using a combination of two protocols: the TRIzol LS protocol (Life Technologies, Carlsbad, USA) and the RNeasy Plus Mini Kit protocol (Qiagen, Hilden, Germany) also including a gDNA removal step (for details see (Watson et al. 2024)). Complementary DNA (cDNA) was obtained by reverse transcription of the RNA (mRNA) using the SuperScript IV First‐Strand kit with oligo(dT) (InvitrogenTM, ThermoFisher Scientific Inc., Carlsbad, USA) following the protocol provided by the manufacturer.

### 4.4 High‐Throughput Amplicon Sequencing

To determine the MHC‐I diversity (number of different MHC‐I alleles per individual) and the MHC‐I gene expression (relative expression of the different MHC‐I alleles within an individual), we used high‐throughput amplicon sequencing (Illumina MiSeq). For gDNA, the best performing primer combination from a previous study, HNalla‐1/R3Ex3b, which amplifies a 246 bp fragment, was used (Mellinger et al. 2023). For all cDNA samples, a newly designed forward primer F3EX3 (5′ GTACAACCWGAGYRGGGGTCT), placed 6 bp upstream compared with HNalla‐1, was used in combination with reverse primer R3Ex3b, which amplified a 252 bp fragment. The primer F3EX3 spans exons 2 and 3 and only has four nucleotides in exon 3; hence, this primer is specifically designed to only amplify MHC‐I exon 3 in cDNA (it cannot amplify exon 3 in gDNA).

All PCR reactions were performed in 25 μL reaction volume using 25–35 ng gDNA or cDNA, 0.5 μM of each primer with Illumina overhangs and 1× Phusion High Fidelity PCR Mastermix with HF Buffer (Thermo Scientific). After an initial denaturation 98°C (30s), the same cycling conditions were used for both primer combination except for annealing temperature: 25 cycles at 98°C (10 s), 62°C for gDNA and 60°C for cDNA (10 s), 72°C (10 s), followed by 72°C for 10 min. Fragments length of PCR products were verified on a 2% agarose gel. For the samples from Kvismaren, PCR products were cleaned up using Agencourt AMPure XP Reagent beads (Beckman Coulter, Indianapolis, USA) following the protocol provided by the manufacturer, but with the following minor changes. A PCR sample to beads ratio of 1:0.8 was used, the two washes was performed with 80% ethanol and the final samples were eluted with 43 μL of ddH_2_O. For the Bulgaria experiment samples, the cleaning was done following the same protocol but on a Microlab NGS Star robot (Hamilton). Eluates of cleaned PCR products were checked on a 2% agarose gel to confirm that only fragments of the correct length were kept. To identify each sample, a dual unique combination of index‐sequences Nextera XT Index Kits v2 (Illumina Inc., San Diego, USA), were added in a second PCR reaction according to manufacturer's protocol. Each 50 μL indexing reaction contained 1× Phusion High Fidelity PCR Mastermix with HF Buffer, 5 μL of each index and 5, 10 or 15 μL of cleaned PCR products depending on the strength of the bands obtained on the agarose gel after cleaning. The cycling conditions consisted of 8 cycles at 98°C (10 s), 62°C (15 s), 72°C (15 s) followed by 72°C for 10 min. Indexed PCR products were cleaned up as mentioned above using a PCR sample to bead ratio of 1:1.12. Cleaned indexed PCR products were check on a 2% agarose gel. The concentration of each sample was determined using the Quant‐iT Picogreen dsDNA Assay Kit (InvitrogenTM, ThermoFisher Scientific Inc., Carlsbad, USA) following the protocol provided by the manufacturer, with changes for adaption to run on 96‐well microplates. 2 μL of cleaned indexed PCR products was diluted with 98 μL 1× TE buffer, and 100 μL PicoGreen was added to each sample. Fluorescence was quantified using a FLUOstar Omega plate reader (BMG Labtech). The concentration of each sample was calculated relative to a standard curve (0.0078 to 0.5 ng/μL) and blank‐corrected. Pools with equimolar DNA amounts were prepared per 96 plate and the concentration of these pools was measured using Qubit dsDNA HS Assay Kits. The quality of the pools (fragment length) was checked on a BioAnalyzer (DNA 1000 Assay Chip, Agilent 2100). Two master pools, one for the Kvismaren data set and one for the Bulgaria experiment data set, were prepared by combining all pools in equimolar DNA amounts and sent to Lund University DNA Sequencing facility for amplicon short‐read sequencing using 300 bp paired‐end Illumina MiSeq sequencing. Replicated samples were included for both the gDNA (43 samples out of 82 in total were replicated) and cDNA (31 samples out of 111 in total were replicated) samples for the Kvismaren data set, and for the Bulgaria experiment (5 samples out of 44 in total were replicated).

### 4.5 Filtering of High‐Throughput Sequencing Amplicons

All HTS amplicons were filtered the same way. First primer sequences and adapters were removed from raw Illumina reads using Cutadapt v2.5 (Martin and Carrington 2013). Samples were demultiplexed and the quality of trimmed reads was assessed using FastQC v0.11.8 (Andrews 2010) and MultiQC v1.7. Next, trimmed reads were filtered with the R packages DADA2 (Callahan et al. 2016) using RStudio 2022.07.1 + 554 (RStudio Team, 2020). DADA2 does most of the filtering for the forward and reverse reads separate. Using the function filterAndTrim the trimmed reads were initially filtered and the settings for maximum number of errors and number of bases trimmed of the end of the reads was adjusting for the forward and reverse read separately, as well as separate for each primer pair combination (Table S2). The error rates were learned with learnErrors, reads were then dereplicated (derepFastq) to combine identical sequences, after which low abundance and low‐quality sequences, that is, likely errors, were clustered to the parental sequences it most likely arose from (with derep). Then the forward and reverse reads were merged (mergePair), and chimeras removed (removeBimeraDenovo). To ensure full coverage any sample with a read depth less than 6000 were removed (one cDNA nestling sample had too low coverage and hence both its gDNA and cDNA samples were removed from further analysis as well as one tissue sample, resulting in the complete removal of samples from that individual). Any sequences with stop codons, or that was not in open reading frame, were deleted. A per amplicon frequency filter was applied to maximize the repeatability among replicates. This threshold was set to 0.0025 for the gDNA, but considerably higher for the cDNA, 0.006, to only keep cDNA alleles that had a reasonable indication of expression (corresponding to keeping alleles with more than 200 reads based on the average total read depth). After the filtering processes, 31 parents, 48 nestlings, 20 1‐year‐old and 9 ≥ 2‐year‐old birds remained in the Kvismaren data set, and nine individuals remained in the Bulgaria experiment data set (Table S3).

### 4.6 Verification of the MHC‐I Genotyping Protocol

For each cDNA sample the read depth of each MHC‐I allele was divided by the total read depth of the HTS amplicon, generating a gene expression profile of the MHC‐I genotype. The proportion of reads is an indicator of relative expression of each MHC‐I allele per individual (the MHC‐I genotype) and we measure how the relative MHC‐I expression varies over time and across tissues within an individual. A limitation of the present study is that we do not measure the absolute expression of the different MHC‐I alleles, since such an estimate could have been directly compared between individuals. The gene expression patterns for the MHC‐I haplotypes are based on the gene expression profile of the cDNA samples (the MHC‐I genotype). MHC‐I haplotypes with less than 10% of the total read depth of the MHC‐I genotype or with only a single MHC‐I allele were excluded from downstream analyses on haplotypes.

The reproducibility of the MHC‐I genotyping protocol, that is, the presence of specific MHC‐I alleles in a sample, was evaluated using two technical replicates for both gDNA and cDNA in 31 individuals (the parents). Presence/absence of each detected MHC‐I allele (percentage identity) was compared between the replicates per individual, separately for gDNA and cDNA. The average reproducibility of the detection of MHC‐I alleles among individuals was 98% for gDNA and 92% for cDNA (*N* = 31 individuals; the threshold for keeping an allele was set to 0.25% for gDNA and 0.6% for cDNA). To evaluate the consistency in the relative expression of alleles within a sample we assessed the repeatability in two independent PCR reactions of the same cDNA sample (technical replicates). To evaluate the consistency in the relative expression of alleles within a sample we calculated Spearman's ρ rank correlations between the proportions of different alleles in two independent PCR reactions of the same cDNA sample (31 technical replicates). The proportion of the different MHC‐I alleles was highly correlated between replicates (r_S_ = 0.94–1.0, mean r_S_ = 0.98, *N* = 31 individuals). Linear regressions were used to investigate if the number of MHC‐I alleles per sample was correlated with total read depth (separately in gDNA and cDNA). The number of MHC‐I alleles per sample (MHC‐I diversity) was not dependent on the total read depth (gDNA: *N* = 79, *r* ^2^ = 0.001, F_(1,77)_ = 0.06 *p* = 0.80; cDNA: *N* = 79, *r* ^2^ = 0.004, F_(1,77)_ = 0.31 *p* = 0.58), showing that MHC‐I diversity is independent of read depth.

### 4.7 Statistical Analyses

To investigate the consistency of expression over time and between tissues, Spearman's rank correlations (ρ) were calculated between different life stages of an individual (nestling, 1‐ vs. ≥ 2‐year‐old birds; *N* = 9 individuals) and between four different tissues within an individual (*N* = 9 individuals). Paired samples Wilcoxon Test was used to check for differences in correlation coefficients between life stages (nestling stage vs. when 1‐year‐old) and ages at adulthood (when 1‐year‐old vs. ≥ 2‐year‐old).

The association in relative expression between parents and offspring or between siblings was estimated as the fixed effect slope in a general linear mixed model (LMM). Family was included as a random effect (both random slope and intercept). LMMs were performed with packages lme4 (Bates et al. 2015) and lmerTest (Kuznetsova et al. 2017) in R 4.4.1 (R Core Team R 2022). MHC‐I expression was log_10_ and Z‐transformed (mean = 0, SD = 1). We report standardized fixed effect slopes as effect size estimates, together with their standard errors. Figures were generated with ggplot2 (Wickham 2016).

## Author Contributions

Helena Westerdahl: conceptualization, data curation, investigation, formal analysis, supervision, writing – original draft, review and editing, funding acquisition. Samantha Mellinger: conceptualization, data curation, formal analysis, investigation, methodology, validation, writing – original draft. Lars Råberg: formal analysis, visualization, writing – review and editing. Anna Drews: data curation, investigation, methodology, validation, writing – review and editing. Bengt Hansson: writing – review and editing, funding acquisition. Pavel Zehtindjiev: methodology, review and editing. Dennis Hasselquist: methodology, writing – review and editing, funding acquisition. All authors gave final approval for publication.

## Funding

The work was supported by the Swedish Research Council (2020‐04285 to HW; 2016‐00689 and 2022‐04996 to BH; 2016‐04391, 2020‐03976 and 2025‐05137 to DH), the European Research Council (ERC) under the European Union's Horizon 2020 research and innovation program (Starting grant 679799 to HW; Advanced grant 742646 to DH), Lunds Djurskyddsfond (to SM), Kungl. Fysiografiska Sällskapet i Lund (to SM), Jörgen Lindströms fond (to SM).

## Ethics Statement

Ethical permit for the capture and collection of blood samples from great reed warblers in Lake Kvismaren has been approved from the Ethical board of Linköping (Dnr 44‐14 (2014–2017), 1633‐2018 (2018–2022), and Dnr 07840‐2023 (2023–2028)). Permit to ring great reed warblers and work in the Kvismaren Nature Reserve have been granted by Länsstyrelsen Örebro (through permit to Kvismare Bird Observatory). Approval for the experiment in Kalimok, Bulgaria was granted by the Bulgarian Ministry of Environment and Waters, permit no. 672/17.03.2016 and 699/23.03.2017. Since 1985, we have studied the great reed warbler population at Kvismaren (Örebro). Each year, all adults and young are ringed, measured, and blood sampled. The great reed warbler study population has increased substantially in size, suggesting that our procedures have little if any impact on performance and survival. Besides generating data for ecological research, this long‐term study has also had a significant positive impact on conservation measures in the Kvismaren Nature reserve. Ethical standards and guidelines of the European Commission were applied in the experiment in Kalimok, Bulgaria, 2018–2020; particular attention has been given to the ‘three Rs’.

## Conflicts of Interest

The authors declare no conflicts of interest.

## Supporting information


**Table S1:** Study set‐up of the 17 great reed warbler families with 1–3 nestlings per clutch that were used when analysing the MHC‐I allele expression patterns over time, including ind_ID, family code, maternal and paternal haplotypes (Fem H2, Fem H1, Male H1 and Male H2), temporal data (t0, t1, t2 and t012), maternal, paternal and offspring haplotypes  (Fem t0, Fem t1, Male t0 and Male t1) and comparisons of sibling maternal and paternal haplotypes (FemHap same, FemHap diff, MaleHap same and MaleHap diff).
**Table S2:** All HTS amplicons were filtered the same way (see methods section), and the following settings were used for the three different data sets (Kvismaren gDNA, Kvismaren cDNA and Bulgaria cDNA).
**Table S3:** The average read depth per sample used in the present study (Illumina MiSeq after filtering) for gDNA and cDNA; in parents and nestlings in the Kvismaren data sets and in the different tissues in the experiment in Kalimok, Bulgaria.

## Data Availability

New nucleotide sequences for the putative alleles described in this study are deposited in GenBank (BankIt3017087, PX552250‐PX552309). Raw read data are deposited in NCBI's Sequence Read Archive under the BioProject PRJNA1366999. Supporting data, including the nucleotide sequences deposited in GenBank, and code are available from the Dryad Digital Repository (https://doi.org/10.5061/dryad.bvq83bkph).
