# Supplementary material for: Static Expression of Major Histocompatibility Complex Genes Across Life‐Stages, Generations and Tissues in a Songbird
Source: Mol Ecol. 2026 Oct 16;35(20):e70590. doi: 10.1111/mec.70590 (PMC13673213; doi:10.1111/mec.70590)
Supplement: Supplementary file 1 [file MEC-35-e70590-s001.docx]

**Table S1**

Study set-up of the 17 great reed warbler families with 1-3 nestlings per clutch that were used when analysing the MHC-I allele expression patterns over time (ind_ID, family code, maternal and paternal haplotypes (Fem H2, Fem H1, Male H1 and Male H2), temporal data (t0, t1, t2 and t012), maternal, paternal and offspring haplotypes (Fem t0, Fem t1, Male t0 and Male t1) and comparisons of sibling maternal and paternal haplotypes (FemHap same, FemHap diff, MaleHap same and MaleHap diff).

**Table S2**

All HTS amplicons were filtered the same way (see methods section), and the following settings were used for the three different data sets (Kvismaren gDNA, Kvismaren cDNA and Bulgaria cDNA).

**Table S3**

The average read depth per sample used in the present study (Illumina MiSeq after filtering) for gDNA and cDNA; in parents and nestlings in the Kvismaren data sets and in the different tissues in the experiment in Kalimok, Bulgaria.

**Table S1.** Study set-up of the 17 great reed warbler families with 1-3 nestlings per clutch that were used when analysing the MHC-I allele expression patterns over time (ind_ID, family code, maternal and paternal haplotypes (Fem H2, Fem H1, Male H1 and Male H2), temporal data (t0, t1, t2 and t012), maternal, paternal and offspring haplotypes (Fem t0, Fem t1, Male t0 and Male t1) and comparisons of sibling maternal and paternal haplotypes (FemHap same, FemHap diff, MaleHap same and MaleHap diff).

| **ind_ID** | **family code** | **Fem H2** | **Fem H1** | **Male H1** | **Male H2** | **t0** | **t1** | **t2** | **t012** | **Fem t0** | **Fem t1** | **Male t0** | **Male t1** | **FemHap same** | **FemHap diff** | **MaleHap same** | **MaleHap diff** |
| --- | --- | --- | --- | --- | --- | --- | --- | --- | --- | --- | --- | --- | --- | --- | --- | --- | --- |
| 3601186 | FAM1 | NA | 1 | 1 | NA | 1 | NA | NA |  |  |  |  |  |  |  | 1 |  |
| 3601187 | FAM1 | NA | 1 | 1 | NA | 1 | NA | NA |  |  |  |  |  |  |  | 1 |  |
| 3601188 | FAM1 | NA | 1 | 1 | NA | 1 | 1 | 1 | 1 |  |  | 1 | 1 |  |  |  |  |
| 3601322 | FAM4 | NA | 1 | NA | 1 | 1 | 1 | NA |  | 1 | 1 | 1 | 1 |  |  |  |  |
| 3601323 | FAM4 | NA | 1 | 1 | NA | 1 | NA | NA |  |  |  |  |  |  |  |  |  |
| 3601324 | FAM4 | 1 | NA | NA | 1 | 1 | NA | NA |  |  |  |  |  |  |  |  |  |
| 3601362 | FAM3a | NA | 1 | 1 | NA | 1 | 1 | 1 | 2 |  |  | 1 | 1 |  |  | 1 |  |
| 3601366 | FAM3a | NA | 1 | 1 | NA | 1 | 1 | 1 | 3 |  |  |  |  |  |  | 1 |  |
| 3617845 | FAM15 | NA | 1 | 1 | NA | 1 | NA | NA |  |  |  |  |  |  | 1 |  |  |
| 3617847 | FAM15 | NA | 1 | NA | 1 | 1 | NA | NA |  |  |  |  |  |  | 1 |  |  |
| 3617849 | FAM15 | 1 | NA | NA | NA | 1 | 1 | NA |  |  |  |  |  |  |  |  |  |
| 3617872 | FAM14 | NA | 1 | 1 | NA | 1 | 1 | NA |  | 1 | 1 | 1 | 1 | 1 |  | 1 |  |
| 3617874 | FAM14 | NA | 1 | 1 | NA | 1 | NA | NA |  |  |  |  |  | 1 |  | 1 |  |
| 3617876 | FAM14 | 1 | NA | NA | 1 | 1 | NA | NA |  |  |  |  |  |  |  |  |  |
| 3617904 | FAM3b | NA | 1 | NA | 1 | 1 | NA | NA |  |  |  |  |  |  | 1 |  |  |
| 3617906 | FAM3b | NA | 1 | 1 | NA | 1 | NA | NA |  |  |  |  |  |  | 1 |  | 1 |
| 3617908 | FAM3b | 1 | NA | 1 | NA | 1 | 1 | NA |  | 1 | 1 |  |  |  |  |  | 1 |
| 3617910 | FAM2 | NA | 1 | 1 | NA | 1 | NA | NA |  |  |  |  |  | 1 | 1 | 1 |  |
| 3617911 | FAM2 | NA | 1 | NA | 1 | 1 | NA | NA |  |  |  |  |  |  | 1 |  |  |
| 3617912 | FAM2 | NA | 1 | 1 | NA | 1 | 1 | NA |  | 1 | 1 | 1 | 1 | 1 |  | 1 |  |
| 3617924 | FAM6 | NA | 1 | 1 | NA | 1 | 1 | 1 | 4 | 1 | 1 | 1 | 1 |  | 1 |  | 1 |
| 3617925 | FAM6 | 1 | NA | 1 | NA | 1 | NA | NA |  |  |  |  |  |  |  |  | 1 |
| 3617927 | FAM6 | NA | 1 | NA | 1 | 1 | NA | NA |  |  |  |  |  |  | 1 |  |  |
| 3617936 | FAM5 | NA | 1 | 1 | NA | 1 | NA | NA |  |  |  |  |  | 1 |  | 1 |  |
| 3617937 | FAM5 | 1 | NA | NA | 1 | 1 | 1 | 1 | 5 | 1 | 1 | 1 | 1 |  |  |  |  |
| 3617938 | FAM5 | NA | 1 | 1 | NA | 1 | NA | NA |  |  |  |  |  | 1 |  | 1 |  |
| 3618050 | FAM7 | NA | 1 | 1 | NA | 1 | NA | NA |  |  |  |  |  |  | 1.1 |  | 1.1 |
| 3618053 | FAM7 | 1 | NA | 1 | NA | 1 | NA | NA |  |  |  |  |  |  | 1.2 |  | 1.1 |
| 3618054 | FAM7 | NA | 1 | NA | 1 | 1 | 1 | 1 | 6 | 1 | 1 | 1 | 1 |  | 1.1 |  | 1.2 |
| 3618055 | FAM7 | 1 | NA | NA | 1 | 1 | 1 | NA |  |  |  |  |  |  | 1.2 |  | 1.2 |
| 3618070 | FAM8 | NA | 1 | 1 | NA | 1 | NA | NA |  |  |  |  |  |  |  |  |  |
| 3618072 | FAM8 | 1 | NA | NA | 1 | 1 | 1 | 1 | 7 | 1 | 1 | 1 | 1 |  |  |  |  |
| 3618091 | FAM9a | NA | 1 | 1 | NA | 1 | 1 | 1 | 8 | 1 | 1 |  |  | 1 |  |  |  |
| 3618092 | FAM9a | NA | 1 | 1 | NA | 1 | NA | NA |  |  |  |  |  | 1 |  |  |  |
| 3618093 | FAM9a | NA | 1 | 1 | NA | 1 | NA | NA |  |  |  |  |  |  |  |  |  |
| 3618185 | FAM13 | NA | 1 | 1 | NA | 1 | 1 | NA |  | 1 | 1 | 1 | 1 | 1 |  | 1 |  |
| 3618186 | FAM13 | NA | 1 | 1 | NA | 1 | NA | NA |  |  |  |  |  | 1 |  | 1 |  |
| 3618187 | FAM13 | 1 | NA | NA | 1 | 1 | NA | NA |  |  |  |  |  |  |  |  |  |
| 3626250 | FAM11 | NA | 1 | 1 | NA | 1 | NA | NA |  |  |  |  |  |  |  |  |  |
| 3626251 | FAM11 | NA | 1 | 1 | NA | 1 | NA | NA |  |  |  |  |  |  |  |  |  |
| 3626253 | FAM11 | 1 | NA | 1 | NA | 1 | 1 | NA |  |  |  |  |  |  |  |  |  |
| 3626255 | FAM10 | NA | 1 | NA | 1 | 1 | 1 | NA |  | 1 | 1 | 1 | 1 |  | 1 |  |  |
| 3626256 | FAM10 | NA | 1 | 1 | NA | 1 | NA | NA |  |  |  |  |  | 1 | 1 | 1 |  |
| 3626257 | FAM10 | NA | 1 | 1 | NA | 1 | NA | NA |  |  |  |  |  | 1 |  | 1 |  |
| 3626293 | FAM9b | 1 | NA | NA | NA | 1 | NA | NA |  |  |  |  |  |  |  |  |  |
| 3626294 | FAM9b | NA | 1 | NA | NA | 1 | 1 | 1 | 9 |  |  |  |  |  |  |  |  |
| 3626296 | FAM9b | 1 | NA | 1 | NA | 1 | 1 | NA |  |  |  |  |  |  |  |  |  |
| 3626298 | FAM12 | NA | 1 | 1 | NA | 1 | 1 | NA |  |  |  |  |  |  |  |  |  |
|  |  |  |  |  |  | 48 | 20 | 9 |  | 11 | 11 | 11 | 11 | 6 | 7 | 7 | 4 |

**Table S2.** All HTS amplicons were filtered the same way (see methods section), and the following settings were used for the three different data sets (Kvismaren gDNA, Kvismaren cDNA and Bulgaria cDNA).

|  | **Kvismaren_gDNA** | **Kvismaren_cDNA** | **Bulgaria_cDNA** |
| --- | --- | --- | --- |
| **MaxN** | 0 | 0 | 0 |
| **maxEE** | 2,3 | 2,3 | 3,4 |
| **truncQ** | 3 | 3 | 4 |
| **truncLen** | 226,175 | 240,175 | Not applied |
| **minLen** | Not applied | Not applied | 100 |
| **rm.phix** | True | True | True |
| **compress** | True | True | True |
| **multithread** | True | True | True |

**Table S3.** The average read depth per sample used in the present study (Illumina MiSeq after filtering) for gDNA and cDNA; in parents and nestlings in the Kvismaren data sets and in the different tissues in the experiment in Kalimok, Bulgaria.

|  | **Average read depth**  **per sample** | **Standard deviation (SD)** | **Number of samples** |
| --- | --- | --- | --- |
|  |  |  |  |
| **Kvismaren_gDNA_parents** | 30009 | 6909 | 31 |
| **Kvismaren_gDNA_ nestlings** | 48641 | 23726 | 48 |
| **Kvismaren_cDNA_parents** | 34752 | 8264 | 31 |
| **Kvismaren_cDNA_nestlings** | 32300 | 5576 | 77 |
| **Bulgaria_cDNA_Blood** | 35188 | 13658 | 9 |
| **Bulgaria_cDNA_Intestine** | 41100 | 19962 | 9 |
| **Bulgaria_cDNA_Liver** | 25998 | 7831 | 9 |
| **Bulgaria_cDNA_Lung** | 34181 | 14477 | 9 |
